# Solitary Angiokeratoma of Oral Mucosa: A Rare Presentation

**DOI:** 10.1155/2013/812323

**Published:** 2013-07-10

**Authors:** Shilpa Kandalgaonkar, Suyog Tupsakhare, Ashok Patil, Gaurav Agrawal, Mahesh Gabhane, Shrikant Sonune

**Affiliations:** Department Oral Pathology & Microbiology, SMBT Dental College & Hospital, Sangamner, Maharashtra 422608, India

## Abstract

Solitary angiokeratoma of oral mucosa is rare entity. The term Angiokeratoma is used to refer to several lesions, whose common denominator is the presence of dilated blood vessels in association with epidermal hyperplasia. Mucosal involvement, including oral cavity is occasionally found either as a component of the systemic variety, cutaneous involvement or isolated oral involvement. Clinically, the lesion is irregular, whitish to dark brown in color, with female predominance. The etiological factors include injury, trauma, or chronic irritation to the wall of a papillary dermis. Histologically, it is characterized by hyperkeratosis, acanthosis, and dilated vascular spaces with or without organizing thrombi in papillary dermis. The vascular spaces are partly or completely enclosed by elongated ret-ridges. Along with this reporting a case of solitary angiokeratoma affecting tongue in a 38-year-old male patient, along with the literature review is presented.

## 1. Introduction

Angiokeratoma is an acquired vascular lesion which is characterized histologically as one or more dilated blood vessels lying directly subepidermally and showing an epidermal proliferative reaction especially acanthosis and hyperkeratosis with dilated capillaries in the papillary dermis [1].

Several clinical types have been described depending on the multiplicity and location of the lesions. They can be divided into localized and systemic types [2]. Mucosal involvement, including the oral cavity, has been described both as localized and systemic types, as a component of Fabry's disease, or as a component of fucosidosis [2–5]. To classify isolated oral mucosal angiokeratomas, other classification systems have been proposed by Ranjan and Mahajan [6].

However, solitary angiokeratomas of the oral mucosa seem to be a rather infrequent occurrence, and very few cases have been reported in the literature. According to the best of our knowledge, since 1997 till date, only 16 cases involving oral cavity have been reported. 

## 2. Case Report

A 38-year-old male patient reported to the Department of Oral Pathology with chief complaint of growth on tip of tongue since last 10 years. The patient was apparently all right 10 years ago when he noticed small painless growth, and then the growth steadily increased in size up to present size involving right side of the tip of the tongue. Sometimes the Patient also experienced bleeding in that area which was associated with trauma during mastication. Bleeding subside, on its own, the patient had never taken any treatment for that growth and not even for bleeding episodes. No abnormality was reveled in his medical, personal histories and general examination. The patient had a habit of tobacco chewing for about 10–15 years.

**Figure 1:**
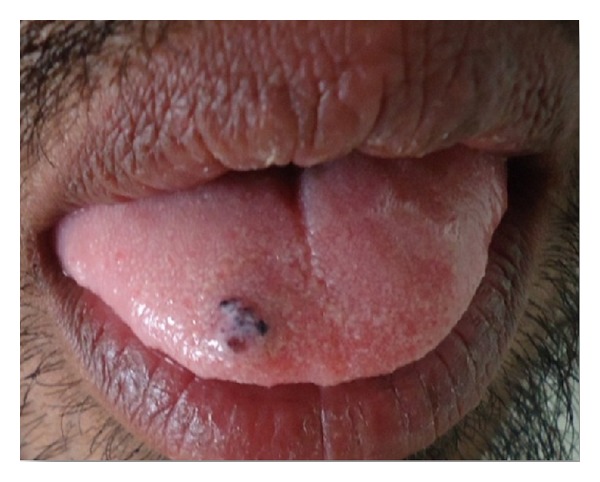
Brownish growth present on tip of tongue.

On clinical examination, it was observed that well-circumscribed sessile growth is present on the dorsal surface of tip of the tongue, and the growth is of approximately 1 × 1 cm in dimension, ovoid in shape. Growth was dark brownish in color with a granular surface texture (Figure 1). On palpation, growth was non tender and rough. No other intra- or extraoral lesions are present.

**Figure 2:**
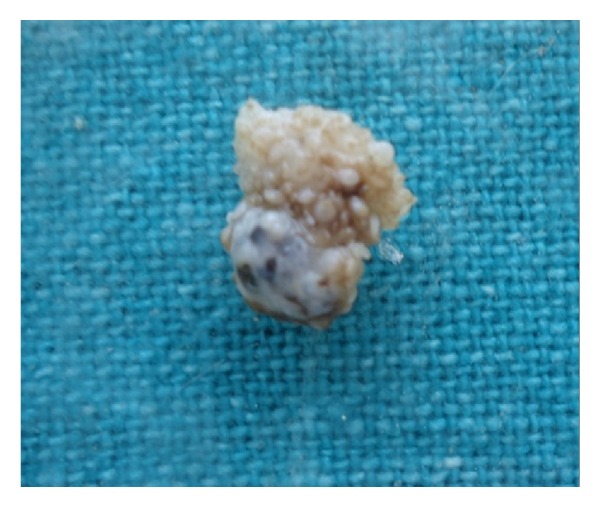
Excised specimen.

After routine hematological investigations, under local anesthesia, the lesion was completely excised and taken for histopathological investigation. The gross specimen is irregular in shape approximately 1 × 1 × 0.5 cm in size, brownish in color, and soft in consistency with rough surface (Figure 2).

**Figure 3:**
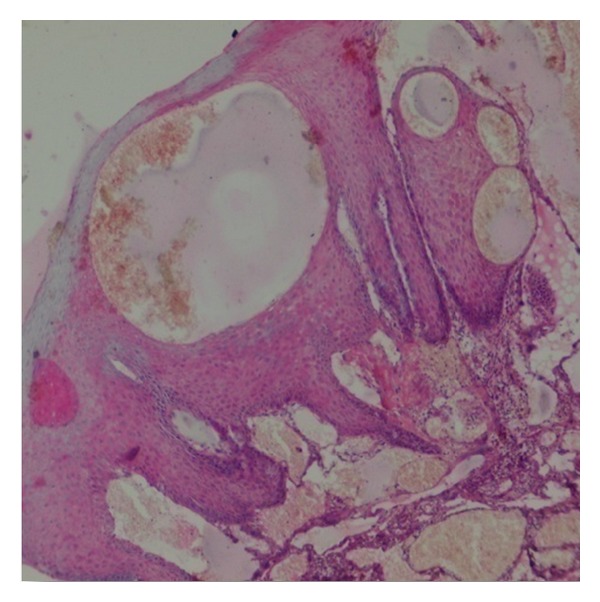
100x magnification.

**Figure 4:**
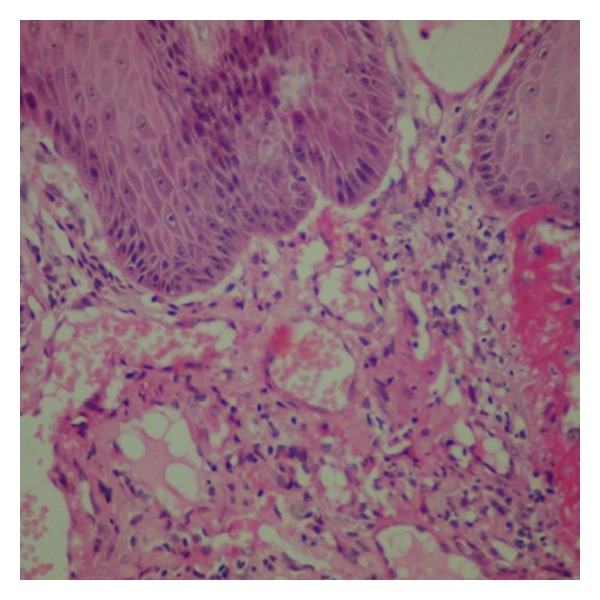
400x magnification.

Histopathologically, parakeratotic stratified squamous epithelium of varying thickness with long slender rete ridges and in some areas large bulbous rete ridges is evident. Papillary connective tissue shows numerous large dilated blood-filled spaces and lined by endothelial cells. Areas of extravasations of blood are also present. Chronic inflammatory cell infiltration around blood vessels and rete ridges is also present. All these features were suggestive of a diagnosis of angiokeratoma (Figures 3 and 4).

**Figure 5:**
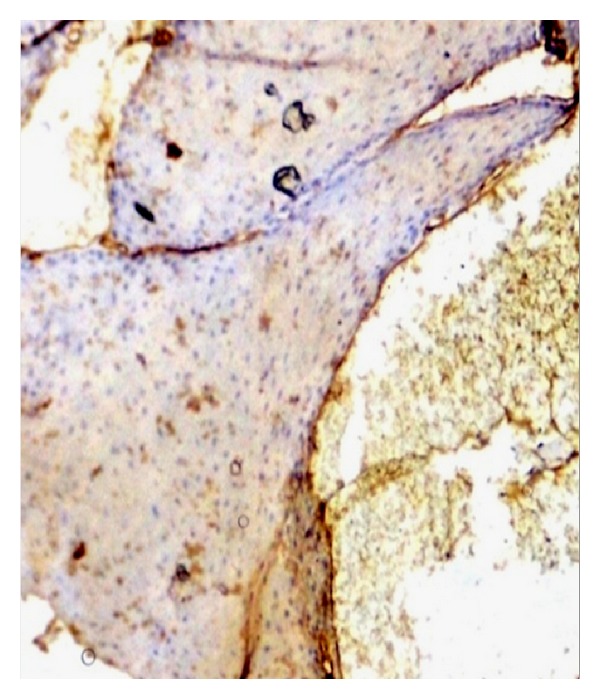
Immunohistochemical profile of the lesion with expression CD34 positive.

For the confirmation of proliferation of blood vessels, CD34 marker was used. The lesion was positive for CD34 (Figure 5). 

After diagnosis, the patient underwent further examinations, and no lesions were found elsewhere in his skin or mucous membranes. The case was considered a solitary angiokeratoma affecting the tongue. In the last followup after six months, the patient was disease-free and asymptomatic. 

## 3. Discussion

Solitary angiokeratoma was first described in 1967 by Imperial and Helwig [7]. These lesions are commonly found on the hips, thighs, buttocks, umbilicus, lower abdomen, scrotum, glans penis, and rarely oral mucosa [8]. Solitary angiokeratomas have been described in the oral cavity, mainly the tongue. Also, one case was also reported on the tonsillar pillar [9]. This lesion seems rather infrequent, and with thorough search, we found only 16 case reports of solitary angiokeratomas affecting oral cavity. 

Pathogenesis of the lesion includes relation to trauma, high venous pressure, or vascular malformation [3]. It is thought that the primary event is vascular ectasia within the papillary dermis just beneath the basement membrane. The epidermal pathological changes seem to be a secondary reaction. It has been reported that the increased proliferative capacity on the surface of vascular malformation related to angiokeratoma [10]. The increase in proliferation of the epithelium is because of the close proximity of the vascular spaces. In case of angiokeratoma, the blood vessels are in close proximity to epithelium, and hence their close proximity to epithelium suggests the secondary proliferation of epithelium [10, 11]. In the present case, histopathology and immunohistochemistry confirm the proposed pathogenesis. 

Review of all the past cases suggests that it is more common in female, but the present case patient was male. The most common site of involvement in the oral cavity is the tongue, the anterior dorsal surface. In present case the site of involvement was also the tongue.

The only clinical problems these lesions can cause are bleeding, discomfort or cosmetic changes [11]. However, most cases were asymptomatic. Therapy has usually been surgical excision in most of the published cases, mainly to discard alternative diagnosis. A recent report has employed diode laser in a 16-year-old woman [12]. Usually, no recurrences have been described [3]. However, few recent cases suggest the recurrence [5]. In the present case, after surgical excision, no recurrence is found after 6-month followup. 

Oral mucosal involvement is a component of angiokeratoma corporis diffusum [8]. If further lesions elsewhere are present, then the possible association with systemic diseases could be expected in widespread cases [3]. Fabry's disease and fucosidosis can be suspected on histopathological grounds by the presence of swollen endothelial cells with a vacuolated cytoplasm in addition to the histology of angiokeratoma [1, 3]. The present case did not show swollen endothelial cells. Also, no other associated lesions were identified. Hence, the present case can be categorized as an isolated solitary angiokeratoma of oral cavity affecting tongue, a recent review by Ranjan and Mahajan. Solitary angiokeratoma of the tongue in adults has proposed a clinical classification for oral angiokeratomas [6].

 Type 1: primary (purely mucocutaneous and not associated with systemic disorders)
   Type 1A, isolated angiokeratomas of the oral cavity
     Type 1As solitary
      Type 1Am multiple
   Type 1B, mucocutaneous angiokeratomas, that is, oral angiokeratomas associated with cutaneous angiokeratomas (e.g., angiokeratomas of vulva/scrotum)
     Type 1Bs solitary
     Type 1Bm multiple
   Type 1C, angiokeratomas occurring simultaneously in oral cavity, skin (e.g., vulva/scrotum), and gastrointestinal mucosa
     Type 1Cs solitary
     Type 1Cm multiple
 Type 2: secondary (as a component of a generalized systemic disorder)
   Type 2A, As a component of Fabry's disease
     Type 2As solitary
     Type 2Am multiple
   Type 2B, as a component of fucosidosis
     Type 2Bs solitary
     Type 2Bm multiple

Considering the same classification, the present case can be categorized as *Type 1As*, that is, *isolated solitary angiokeratoma*.

The main differential diagnosis on histopathological grounds was lymphangioma, to exclude the diagnosis and to confirm the proliferating blood vessels. Immunohistochemical staining is implied. In the previous literature, antigens used were CD31, CD34, and LYVE-1 (lymphatic vessel endothelial hyaluronan), and CD31 and CD34 were found positive and LYVE-1 (lymphatic vessel endothelial hyaluronan) was negative [3]. In the present case, antigen used was CD34. CD34 antigen that was used is considered as a reliable marker for the proliferating blood vessels. CD34 was positive in the present case which confirms the proliferating blood vessels. 

The differential diagnosis of angiokeratoma is important because of its similarity to some other lesions [5, 12]. Other vascular lesions like hemangioma, and lymphangioma can be ruled out with the help of histopathological investigation. In case of hemangioma, small capillary lined by single layer of endothelial cells supported by connective tissue stroma is seen [13]. Also, endothelial cell proliferation is also noted. These blood vessels are completely lain within the connective tissue, while in case of angiokeratoma, blood vessels are supported by epithelium and lie very close to the epithelium [12].

In case of lymphangiomas, multiple intertwining lymph vessels lie very close to the epithelium and are seen also in papillary connective tissue. Presence of blood-filled spaces and endothelial lining also helped to differentiate angiokeratoma from lymphangiomas [12].

Angiokeratoma can be clinically confused with the aggressive lesions like malignant melanoma, especially in case of angiokeratoma when the vessels are thrombosed [14]. Histopathological examination can only differentiate angiokeratoma from malignant melanoma [1]. In malignant melanoma presence of atypical melanocytes, in clusters or groups, also singly placed. These cells show prominent nuclei often with prominent nucleoli [13]. Such appearances are not seen in case of angiokeratoma.

## 4. Conclusions

Oral angiokeratomas of the oral cavity are rare tumors. Although they can appear as isolated lesions, their presence should prompt further investigations to rule out systemic disease.
